# Preparation of Clathrin-Coated Vesicles From *Arabidopsis thaliana* Seedlings

**DOI:** 10.3389/fpls.2018.01972

**Published:** 2019-01-09

**Authors:** Niccolò Mosesso, Tobias Bläske, Marie-Kristin Nagel, Michael Laumann, Erika Isono

**Affiliations:** ^1^Chair of Plant Physiology and Biochemistry, Department of Biology, University of Konstanz, Konstanz, Germany; ^2^Electron Microscopy Centre, Department of Biology, University of Konstanz, Konstanz, Germany

**Keywords:** clathrin coated vesicles, density fractionation, *Arabidopsis thaliana*, negative staining, scanning electron microscopy

## Abstract

Clathrin coated vesicles (CCVs) mediate endocytosis of plasma membrane proteins and deliver their content to the endosomes for either subsequent recycling to the plasma membrane or transport to the vacuole for degradation. CCVs assemble also at the trans-Golgi network (TGN) and is responsible for the transport of proteins to other membranes. Oligomerization of clathrin and recruitment of adaptor protein complexes promote the budding and the release of CCVs. However, many of the details during plant CCV formation are not completely elucidated. The analysis of isolated CCVs is therefore important to better understand the formation of plant CCVs, their cargos and the regulation of clathrin-mediated transport processes. In this article, we describe an optimized method to isolate CCVs from *Arabidopsis thaliana* seedlings.

## Introduction

Plants regulate their development to the changing environment by sensing and responding to environmental signals. Plasma membrane associated receptors, transporters and lipids play an important role in coordinating extracellular signals with intracellular responses. The regulation of plasma membrane proteins is therefore critical for optimal plant development and growth. Besides transcriptional control, endocytosis and subsequent endosomal trafficking of plasma membrane proteins is also essential for correct environmental responses (Paez Valencia et al., 2016). The endocytosis of a membrane protein can be either dependent on clathrin (clathrin-mediated endocytosis [CME]) or independent (McMahon and Boucrot, 2011; Sandvig et al., 2011, 2018; Baisa et al., 2013; Mayor et al., 2014; Robinson, 2015). Clathrin build a coat around the endocytosed vesicle to form a clathrin coated vesicle (CCV). The clathrin coat is formed by multiple units composed of three clathrin heavy chains (CHCs) and three clathrin light chains (CLCs) that oligomerize together to form three-legged structures called *triskelia*.

Several accessory proteins, in concert with the heterotetrameric adaptor complex ADAPTIN PROTEIN 2 (AP2), link the clathrin coat to the plasma membrane bilayer and recognize specific cargo proteins for endocytosis. Another important adaptor complex in *Arabidopsis* is the eight core-component complex TPLATE (TPC) that is important for clathrin recruitment at the plasma membrane and for the initiation of CME (Van Damme et al., 2011; Gadeyne et al., 2014). Once the clathrin adaptors have selected the cargos and the clathrin coat is assembled to a clathrin coated pit (CCP), the local membrane curvature resolves into a CCV upon scission from the plasma membrane. Once CCVs are released into the cytoplasm, they undergo uncoating before delivering their cargos to the trans-Golgi network (TGN)/early endosomal compartment (EE) (Dettmer et al., 2006). The endocytosed cargos can either be recycled back to the plasma membrane or transported to the vacuole for degradation (Viotti et al., 2010; Paez Valencia et al., 2016; Reynolds et al., 2018). Clathrin is also recruited onto the TGN by adaptor complexes AP1 and AP3. CCVs can be generated from the surface of the TGN and are responsible for post-Golgi trafficking to the vacuole and for transport to the plasma membrane (Robinson and Pimpl, 2014; Rosquete et al., 2018).

Research in the past decade has shown that clathrin-mediated transport is essential for important physiological processes in plants. Although many regulatory factors have been identified, we still lack in-depth understanding on the nature of CCV cargos and the exact mechanisms of clathrin-dependent vesicle trafficking. One limitation, so far, was the difficulty to isolate and analyze CCVs from different *Arabidopsis* mutants. CCVs were originally isolated and purified from pig brains in which they are highly abundant (Pearse, 1975). In the original protocol, CCVs were enriched by differential centrifugation followed by linear sucrose density gradient centrifugations. This protocol was further improved by the use of isotonic linear D_2_O/Ficoll gradient (Pearse, 1980, 1982). Two protocols for CCV isolation from suspension-cultured carrot cells and from *Pisum sativum* cotyledons were described (Depta and Robinson, 1986; Harley and Beevers, 1989). Both involve sucrose step-gradient centrifugation and linear D_2_O/Ficoll gradient centrifugation. Later, a method to isolate CCVs from *Arabidopsis thaliana* suspension-cultured cells was established based on these protocols (Reynolds et al., 2014).

In this article, we describe a modified method for CCV isolation from *Arabidopsis* seedlings. The protocol enables to circumvent the time-consuming steps required for the preparation of *Arabidopsis* suspension-cultured cells and allows isolation of CCVs directly from entire *Arabidopsis* seedlings.

## Materials and Methods

### Plant Materials and Growth Conditions

All experiments were performed with *A. thaliana* Columbia-0 (Col-0). *ARA7pro:mRFP-*ARA7 line has been described previously (Inada et al., 2016). The seeds were sterilized using a 1% NaOCl solution and then plated on Murashige and Skoog growth medium with Gamborg B5 vitamins (Duchefa Biochemie) supplemented with 1% sucrose. Seedlings were grown vertically in long day (16/8 h photoperiod) at 110 μmol m^-2^⋅s^-1^ light intensity at 21°C.

### Reagents, Solutions and Buffers

All the solutions were prepared using ddH_2_0 and chemicals of analytical quality based on the previous publication (Reynolds et al., 2014) with some modifications. Solutions indicated with an asterisk (^∗^) are supplemented with the following protease inhibitors: 500 μM PMSF and 7.3 μM pepstatin A, 2.1 μM leupeptin-hemisulfate and 1 mM DTT.

• 500 mM PMSF and 7.3 mM pepstatin A solved in DMSO. Store at -80°C in 200 μL aliquots.
• 2.1 mM leupeptin-hemisulfate in ddH_2_O. Store at-80°C in 200 μL aliquots.
• 1 M dithiothreitol (DTT) in ddH_2_O. Store at -80°C in 200 μL aliquots.
• Clathrin Isolation Buffer (CIB), pH 6.4: 100 mM MES monohydrate [2-(N-morpholino) ethanesulfonic acid monohydrate], 0.5 mM MgCl_2_ (magnesium chloride), 3 mM EDTA (ethylenediaminetetraacetic acid), 1 mM EGTA (ethylene glycol tetraacetic acid). pH adjusted with 5 N KOH; filter (pore size ≤ 0.4 μm) and store at 4°C until use. Prepare 150 mL and use to prepare CIB^∗^ and the sucrose solutions^∗^.
• CIB^∗^: Prepare 100 mL.
• Sucrose solutions^∗^: Use analytical grade sucrose to prepare 2.5 mL, 11 mL, and 7 mL of 50%, 25%, and 12% w/v sucrose solutions in CIB, respectively. Can be prepared a day before as sucrose takes time to dissolve at higher concentrations. Filter (pore size ≤ 0.4 μm) and store at 4°C until use. Supplement the sucrose solutions with the protease inhibitors before use (see above).
• 90% D_2_O/30% Ficoll solution^∗^: Prepare 15 mL of 90% w/v D_2_O, 30% w/v Ficoll, 100 mM MES monohydrate, 0.5 mM MgCl_2_, 3 mM EDTA pH 8.0, 1 mM EGTA pH 8.0. Incubate 1 h at 60°C while mixing. Adjust pH to 6.4 with 10 N NaOH and bring to the final volume with ddH_2_O. Can be prepared a day before. Store at 4°C until use. Supplement the solution with the protease inhibitors before use (see above).
• 18% D_2_O/5% Ficoll solution^∗^: Prepare 15 mL of 18% w/v D_2_O, 5% w/v Ficoll, 100 mM MES monohydrate, 0.5 mM MgCl_2_, 3 mM EDTA pH 8.0, 1 mM EGTA pH 8.0. Incubate 1 h at 60°C while mixing. Adjust pH to 6.4 with 10 N NaOH and bring to the final volume with ddH_2_O. Can be prepared a day before. Store at 4°C until use. Supplement the solution with the protease inhibitors before use (see above).
• 5x SDS sample Buffer (Laemmli, 1970): 310 mM Tris–HCl pH 6.8, 50% (w/v) glycerol, 10% (w/v) SDS, 0.5% (w/v) Bromophenol blue, 3.5% (w/v) β-mercaptoethanol.
• 4% w/v OsO_4_ (Osmium tetraoxide). Make 10 μL aliquots and store at -30°C.
• Methylamine tungstate (Nano-W, Nanoprobes) ready-to-use 2% solution in water at pH 6.8. Store at 4°C. The solution is stable for up to 1 year after first use.

### Equipment

• Laboratory standard refrigerated centrifuge (Eppendorf^TM^ Centrifuge 5424R or equivalent) and accompanying rotor (FA-45-24-11 or equivalent).
• Refrigerated high-speed centrifuge (Eppendorf^TM^ Centrifuge 5804R or equivalent) and accompanying rotor (FA-45-6-30 or equivalent).
• Refrigerated high-speed centrifuge (Beckman Coulter Avanti^TM^ J-25 or equivalent) with a rotor for 50 mL disposable tubes (JA-14 and Beckman Coulter conical tube adapters, or equivalent).
• High-speed ultracentrifuge (Beckman Coulter Optima^TM^ LE-80K Ultracentrifuge or equivalent) compatible with different rotors and tubes for differential and density gradient ultracentrifugation (SW 40 Ti swinging-bucket rotor and Type 70 Ti fixed-angle titanium rotor, or equivalent).
• Ultra-Clear centrifuge tubes (14 × 95 mm) for density gradient centrifugations (Beckman Coulter).
• Polycarbonate tubes with Cap Assembly for use in Ultracentrifuge Rotors (Beckman Coulter).
• Clean mortar and pestles.
• Disposable sterile needles 1.6 × 25 mm – 16 G × 1″.
• Refractometer.
• TEM square mesh support grids, 200 mesh, nickel (Plano GmbH).
• Non-magnetic steel forceps.
• Scanning electron microscope (Zeiss, Auriga Crossbeam).

### Methods

For the isolation of CCVs, membranes are first enriched from plant total extracts by ultracentrifugation. The membrane fraction is then separated on a sucrose step gradient and further separated on a D_2_O/Ficoll linear gradient. Medium density fractions are subsequently subjected to ultracentrifugation to obtain a pellet that contains the isolated intact CCVs.

**FIGURE 1:**
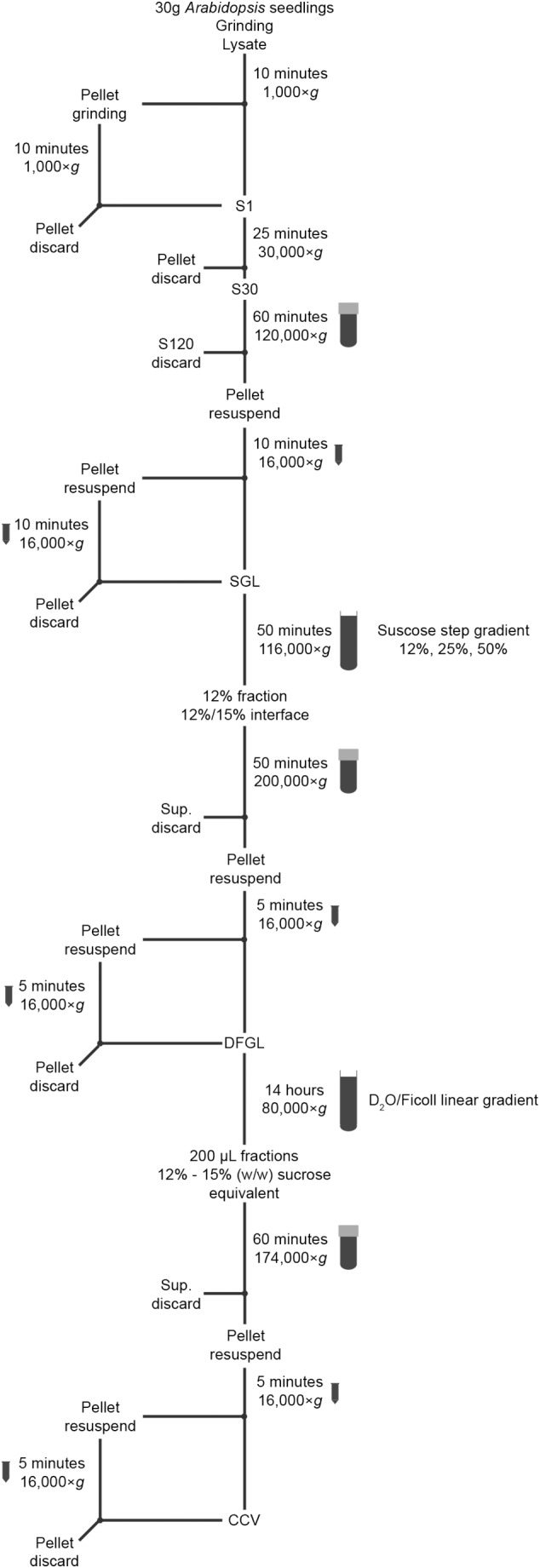
Schematic representation of the CCV-isolation procedure.

Keep all the solutions, mortars and pestles at 4°C overnight and pre-chill centrifuges prior to use. Prepare solutions indicated with an asterisk (^∗^) immediately before use. The overview of the whole procedure is schematically presented in Figure 1. The isolation of CCVs will take 2 days including the preparation of solutions and all centrifugation steps. The subsequent SEM analysis should be carried out with freshly isolated CCVs. If this is not possible, the isolated CCVs can be stored overnight at 4°C and then processed for the SEM analysis. The preparation of the SEM samples take half a day including the imaging. The prepared grids for SEM analysis can be kept overnight in a desiccator with vacuum applied and imaged the following day.

### Preparation of D_2_O/Ficoll Gradient for Ultracentrifugation

(1) Prepare 15 mL 90% D_2_O/30% Ficoll solution^∗^ and 15 mL 18% D_2_O/5% Ficoll solution^∗^.
(2) Gently add 7 mL of 90% D_2_O/30% Ficoll solution^∗^ to each of the two Ultra-Clear centrifuge tubes (14 × 95 mm).
(3) Slowly fill up the two Ultra-Clear centrifuge tubes (14 × 95 mm) with 7 mL of 18% D_2_O/5% Ficoll solution^∗^ without disturbing the bottom layer.
Note: The ultracentrifuge tubes must be completely filled up. If the amount of solution is not enough, add more 18% D_2_O/5% Ficoll solution^∗^.
(4) Close the centrifuge tubes with a plastic film and lay them down very carefully on a small tray. Keep the tubes horizontally in a stable position at 4°C for at least 6 h.
Note: Alternatively, the gradient can be generated using a gradient maker.

### CCV Purification

(1) Prepare 30 g of 7-days-old seedlings. In a cold room, grind 5 g of the seedlings in a clean mortar with 5 mL ice cold CIB^∗^. Collect the lysate and keep in a 50 mL tube on ice. Repeat until all seedlings are processed. Distribute the lysate equally into two 50 mL conical tubes. Save 500 μL in a 1.5 mL tube and label as “Lysate” for later analysis.
(2) Centrifuge the lysate for 10 min at 1,000 ×*g* at 4°C. Carefully collect the supernatant with a 25 mL disposable sterile pipette into a fresh 50 mL conical tube and keep on ice.
(3) To increase the yield of CCVs, transfer the pellets from the two tubes to a mortar and grind the pellets with 6 mL CIB^∗^ and collect the lysate in a fresh 50 mL tube. Centrifuge the lysate for 10 min at 1,000 × *g* at 4°C. Carefully collect the supernatant and add it to the previously obtained supernatant from step 2. Save 500 μL of sample in a 1.5 mL tube and label as “S1” for later analysis.
(4) In a cooled high-speed centrifuge, centrifuge the supernatant for 25 min at 30,000 ×*g* at 4°C. Save 500 μL of sample in a 1.5 mL tube and label as “S30” for later analysis.
(5) Transfer the supernatant to two 26.3 mL polycarbonate ultracentrifuge tubes and centrifuge for 1 h at 120,000 ×*g* at 4°C.
(6) While waiting for the centrifugation, prepare the tubes for the sucrose step gradient. Prepare 2.5 mL 50% (w/v) sucrose solution^∗^, 11 mL 25% (w/v) sucrose solution^∗^ and 7 mL 12% (w/v) sucrose solution^∗^.
(7) Prepare two Ultra-Clear centrifuge tubes (14 × 95 mm), and first layer gently 1 mL of 50% (w/v) sucrose solution^∗^. Slowly add 5 mL of 25% sucrose solution^∗^ and subsequently add 3 mL of 12% sucrose solution^∗^ into each tube without disturbing the other layers. Keep the tubes in a tube rack at 4°C until use.
(8) Discard the supernatant.
(9) Resuspend the two pellets in 1 mL CIB^∗^ each. Transfer the homogenate into two 1.5 mL tubes and centrifuge for 10 min at 16,000 ×*g* at 4°C.
(10) Transfer the supernatant to a 15 mL conical tube. Resuspend the pellets remaining in the 1.5 mL tubes with 1.5 mL of CIB^∗^ each. Centrifuge 10 min at 16,000 ×*g* at 4°C. Pool the supernatant together. This should yield a final volume of approximately 5 mL. Save 200 μL in a 1.5 mL tube and label as “SGL” for “sucrose gradient load” for later analysis.
(11) Carefully layer 2.4 mL of the supernatant on each of the sucrose step gradient prepared in step 7. Centrifuge 50 min at 116,000 ×*g* at 4°C.
Note: During this step, carefully handle the ultracentrifuge tubes to avoid disturbing the gradient. Select slow acceleration and slow deceleration.
(12) After the centrifugation, collect the 12% (w/v) layer and the 12%/25% (w/v) interface. Transfer each of the collected layers into a fresh 26.3 mL polycarbonate ultracentrifuge tube.
Note: Avoid taking the 25% (w/v) fraction to prevent contamination with denser membrane structures.
(13) Dilute the content of both 26.3 mL polycarbonate ultracentrifuge tube with 13 mL CIB^∗^ each. Sucrose concentration should be less than 5% (w/v). Centrifuge 50 min at 200,000 ×*g* at 4°C.
Note: Measure the refractive index of the diluted content using a refractometer. Add more CIB^∗^ to dilute the sucrose concentration to below 5% (w/v), if necessary.
(14) Discard the supernatant and resuspend each of the two pellets in 300 μL CIB^∗^. Transfer the homogenate into two 1.5 mL tubes and centrifuge for 5 min at 16,000 ×*g* at 4°C.
(15) Transfer the supernatant to a fresh 1.5 mL tube. Resuspend the pellets left after step 14 in 50 μL CIB^∗^ each. Centrifuge 5 min at 16,000 ×*g* at 4°C. Add the supernatant to the 1.5 mL tube, which should result in a final volume of approximately 700 μL. Save 100 μL in a 1.5 mL tube and label as “DFGL” for “D_2_O/Ficoll gradient load” for later analysis.
(16) Carefully remove 500 μL of the D_2_O/Ficoll gradient prepared in 2.4.1.
Note: Skip this step if linear gradient was prepared with a gradient mixer.
(17) Layer 300 μL of the supernatant obtained in step 15 on each D_2_O/Ficoll gradient without disturbing the gradient. Centrifuge 14 h at 80,000 × *g* at 4°C.
Note: During this step, carefully handle the ultracentrifuge tubes to avoid disturbing the gradients. Select slow acceleration and slow deceleration.
(18) After the centrifugation, keep one of the ultracentrifuge tubes on ice and make a hole in the bottom of the other tube with a G16 needle. Fractionate the entire gradient in 0.2 mL aliquotes by collecting the drops.
(19) Repeat step 18 with the remaining tube.
(20) Measure the sucrose percentage (w/w) equivalent of each fraction from one gradient with a refractometer. CCVs are present in the fractions between 12% and 15% (w/w) sucrose equivalent (refractive index [at 20°C] = 1.35093 – 1.35568). Collect the corresponding fractions and transfer them to a fresh 26.3 mL polycarbonate ultracentrifuge tube.
(21) Repeat step 20 with the fractions from the second gradient.
(22) Dilute the content of the 26.3 mL polycarbonate ultracentrifuge tubes with 8 mL CIB^∗^ each. Sucrose percentage (w/w) equivalent of the solution should be less than 5 % (w/v). Centrifuge 1 h at 174,000 ×*g* at 4°C.
Note: measure the refractive index of the diluted content using a refractometer. Add more CIB^∗^ to dilute the sucrose (w/w) equivalent concentration to below 5% (w/v), if necessary.
(23) Discard the supernatant and resuspend the pellet in one 26.3 mL polycarbonate ultracentrifuge tubes with 70 μL CIB^∗^. Transfer the homogenate to the other 26.3 mL polycarbonate ultracentrifuge tube and resuspend the pellet. Transfer the resulting homogenate in 1.5 mL tubes and centrifuge 5 min at 16,000 ×*g* at 4°C.
Note: Remove the supernatant completely with a pipette without disturbing the pellet.
(24) Transfer the supernatant to a fresh 1.5 mL tube. Resuspend the pellet left after step 23 in 10 μL CIB^∗^. Centrifuge 5 min at 16,000 ×*g* at 4°C.
(25) Transfer the supernatant to the tube from step 24. The total volume should be 80 μL. This contains the enriched CCVs. Save 70 μL in the tube and label it as “CCV” for later analysis and the remaining 10 μL for electron microscope analysis.

### Negative Stain and SEM Analysis

(1) Glow discharge carbon-coated 200-mesh nickel grids.
(2) Immobilize carbon-coated 200-mesh nickel grids by clasping with non-magnetic negative steel forceps.
(3) In an appropriate fume hood, on a bit of plastic film mix one part of the CCV fraction and three part of 4% (w/v) OsO_4_ solution (e.g., 1 μL CCV fraction and 3 μL OsO_4_ solution). Mix thoroughly by pipetting.
(4) Carefully deposit 1 μL of the CCV mixture (step 3, above) on the face of each of the immobilized grids. Let dry under the fume hood.
(5) Place a droplet of Nano-W ready-to-use 2% solution onto a plastic film. Transfer 10 μL of Nano-W ready-to-use 2% solution on each of the dried mixture-coated grids. Incubate for 1 min. Using a wedge of Whatman paper, wick away excess fluid until the grids are nearly dry. Let dry under the fume hood.
Note: Avoid touching the surface of the grids with paper. Accordingly, touch the edges of the Nano-W ready-to-use 2% solution droplet and remove as much solution as possible.
(6) Image the grids using a scanning electron microscope in scanning transmission electron microscope (STEM) mode with 30 kV. A 25,000 × magnification (calculated for a Polaroid 545) is sufficient to distinguish CCVs from uncoated vesicles.

### Immunoblot

Samples collected during the purification were heated for 5 min at 65°C in 1 × Laemmli buffer and analyzed by immunoblot using antibodies against specific organelles/cellular compartments. SDS/PAGE and immunoblotting were performed according to standard protocols. Antibodies used for immunoblotting were anti-CHC (Agrisera), anti-BIP2 (Agrisera), anti-Sec21p (Agrisera), anti-TOC75 (Agrisera), anti-V-ATPase (Agrisera), anti-RFP (clone 6G6; Cromotek).

## Results and Discussion

**FIGURE 2:**
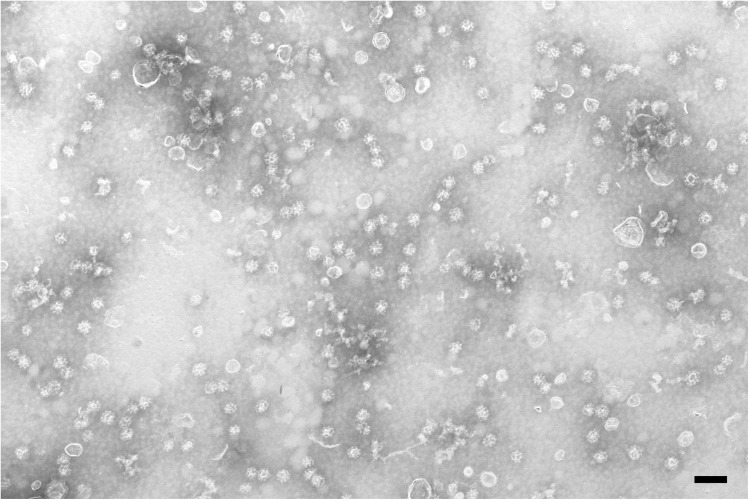
Negative STEM micrograph of the final CCV fraction prepared from 7-day-old wild-type seedlings. Scale bar: 200 nm.

To determine the presence of intact CCVs, we examined isolated CCVs from 7-day-old wild-type seedlings under a scanning electron microscope. Negative staining and scanning electron microscopy analysis of the sample showed the presence of intact CCVs (Figure 2). Since uncoated vesicles were also present, we determined the relative amount of CCVs to the total number of intact membrane structures. We observed 60.3% CCVs in two independent experiments (*n* = 1714 vesicles).

**FIGURE 3:**
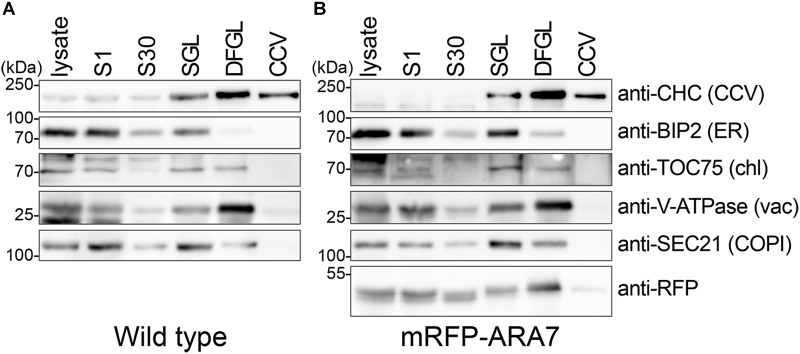
CCVs were prepared from total plant extract of 7-day-old wild type **(A, B)** mRFP-ARA7 seedlings. Samples were collected during the procedure of CCV isolation and subjected to Western blot analyses using antibodies against CHC, mRFP, and various subcellular organelle marker proteins. CCV, CCV-containing fraction; DFGL, linear D_2_O/Ficoll gradient load; S1, supernatant after 1,000 ×*g* centrifugation; S30, supernatant after 30,000 ×*g* centrifugation; SGL, sucrose step gradient load. The following antibodies were used as organelle- or compartment-specific markers: anti-V-ATPase (vacuole), anti-TOC75 (chloroplast), anti-BiP2 (ER), anti-Sec21p (COPI).

To further evaluate the purity of CCVs we analyzed the obtained samples by immunoblotting using an anti-CHC antibody and antibodies specific to intracellular organelles (Figure 3A). CHC was enriched in the final fraction, whereas other marker proteins for chloroplast (TRANSLOCON ON THE OUTER CHLOROPLAST MEMBRANE 75 [TOC75]), ER (BINDING PROTEIN 2 [BIP2]), Coat protein complex I (COPI) vesicles (SECRETORY21 [SEC21p]), and vacuole (V-ATPase SUBUNIT E1 [VHA-E1]) were not enriched in the CCV fraction. Together, these results suggest that CCVs were successfully isolated without major contamination of other organelles.

We conducted the same experiment using 7-d-old seedlings expressing the late endosome marker mRFP-ARABIDOPSIS RAB GTPase 7 (ARA7) under the control of its native promoters (Inada et al., 2016). When analyzed with the same antibodies used in Figure 3A, mRFP-ARA7 fractionated differently (Figure 3B). Although, faint signals of mRFP-ARA7 were still visible in the CCV enriched fraction, in contrast to CHC, mRFP-ARA7 was not enriched when compared to the total lysate. Although there are reports of flat clathrin patches on the surface of endosomes (Smith and Farquhar, 1966; Prekeris et al., 1998; Prekeris et al., 1999; Raiborg et al., 2001, 2002; Sachse et al., 2002; Williams and Urbe, 2007), to date there is no evidence of CCV formation from ARA7-positive late endosomal compartments.

## Conclusion

The method presented here is suitable for the isolation of CCVs from *Arabidopsis* seedlings grown under standard conditions. This enables the quantitative and morphological analyses on CCVs and CCV-associated proteins from different *Arabidopsis* mutant backgrounds or upon treatment with compounds of interest. We verified the successful enrichment and isolation of intact CCVs applying this method by immunoblotting and scanning electron microscopy.

## Author Contributions

NM, TB, M-KN, and EI optimized the method. NM, TB, and ML conducted negative staining and analysis by SEM. NM and EI wrote the manuscript with the help of ML.

## Conflict of Interest Statement

The authors declare that the research was conducted in the absence of any commercial or financial relationships that could be construed as a potential conflict of interest.
